# Analysis of risk factors for herb-induced liver injury: a retrospective study from China

**DOI:** 10.3389/fphar.2026.1766238

**Published:** 2026-02-18

**Authors:** Pengchong Wang, Xinyan Liang, Guangyu Cheng, Qimeng Fan, Qing Li, Fengshou Dai, Yajun Liu, Zhenzhou Jiang, Tianjiong Luo, Jingwen Hu, Wenliang Dun

**Affiliations:** 1 Nanjing Hospital of Chinese Medicine Affiliated to Nanjing University of Chinese Medicine, Nanjing, Jiangsu, China; 2 Department of Clinical Pharmacy, Nanjing Hospital of Chinese Medicine Affiliated to Nanjing University of Chinese Medicine, Nanjing, Jiangsu, China; 3 School of Pharmacy, Nanjing University of Chinese Medicine, Nanjing, Jiangsu, China; 4 School of Basic Medicine and Clinical Pharmacy, China Pharmaceutical University, Nanjing, Jiangsu, China; 5 School of Public Health, Southeast University, Nanjing, Jiangsu, China; 6 Department of Geriatrics, Nanjing Hospital of Chinese Medicine Affiliated to Nanjing University of Chinese Medicine, Nanjing, Jiangsu, China; 7 Department of Gastroenterology, Jiangsu Province Hospital of Chinese Medicine, Nanjing, Jiangsu, China; 8 Key Laboratory for Drug Quality Control and Pharmacovigilance, Ministry of Education, China Pharmaceutical University, Nanjing, Jiangsu, China; 9 Department of Pharmacy, Nanjing Hospital of Chinese Medicine Affiliated to Nanjing University of Chinese Medicine, Nanjing, Jiangsu, China

**Keywords:** hepatic dysfunction, herb, herb-induced liver injury, immune response, risk factors

## Abstract

**Objective:**

To analyse the clinical and laboratory characteristics of patients experiencing liver injury following herbal medication use, and to identify herb-induced liver injury in patients with risk factors.

**Methods:**

This retrospective study included patients admitted to Nanjing Hospital of Chinese Medicine affiliated to Nanjing University of Chinese Medicine, between December 2014 and December 2023 who developed liver injury following herbal medicine consumption. The cohort was divided into a training set and an validation set. Patients admitted to Jiangsu Province Hospital of Chinese Medicine between January 2024 and March 2025 who developed liver injury following herbal medicine consumption were included as the test set. Predictor variables were screened using univariate analysis and binary logistic regression, and predictive models were constructed for risk factors in patients with herb-induced liver injury versus non-drug-induced liver injury. Evaluate model performance using multiple assessment metrics, including AUC and DCA.

**Results:**

The analysis included 3,914 patients with abnormal liver function who had a history of herbal medicine use, of whom 176 were assessed as having herb-induced liver injury through the RUCAM causality assessment. Research has found that ALP is an independent risk factor distinct from WILI. Incorporate risk factors for both the HILI group and NON-DILI group: age, alcohol, urea, platelet distribution width, and monocyte ratio. Construct a predictive model and evaluate its performance. The model demonstrates favourable performance in terms of AUC and DCA across both the training and validation sets.

**Conclusion:**

Compared with patients suffering from non-drug-induced liver injury, those of advanced age, with concomitant hepatic metabolic dysfunction, or underlying immune disorders exhibit a significantly higher risk of developing herb-induced liver injury.

## 1 Introduction

Drug-induced liver injury (DILI) is a severe adverse drug reaction with significant clinical harm. Numerous marketed drugs and dietary fibre supplements may potentially cause liver damage, which is also a primary factor leading to the withdrawal of medicinal products from the market following their launch (Rani et al., 2024; Onakpoya et al., 2016). Herbal remedies are widely perceived as “natural” and “safe”, exposing misconceptions in public health education regarding their use. This perception may lead to the misuse or inappropriate application of herbal remedies, complicating diagnosis (Teschke, 2014). Epidemiological investigations reveal that the incidence rate of DILI in China stands at 23.80 per 100,000 population, with herb-induced liver injury (HILI) accounting for 25% of all liver injury cases (Yang Y. et al., 2022). This incidence rate is higher than that observed in western countries (Shen et al., 2019; Bessone et al., 2022). It is evident that herbal medicines pose a serious threat to human health globally, particularly in China.

HILI is characterised by its insidious onset and difficulty in identification, with accurate diagnosis remaining a challenging issue for scholars worldwide. The occurrence of herb-induced liver injury is influenced by multiple factors, including the herbal preparation itself, the host individual, environmental conditions, polypharmacy, and the species and quality of the herbal materials used in clinical practice, all of which may increase the risk of hepatotoxicity (Wang et al., 2018). HILI is typically unrelated to the dosage of the drug, the majority of patients with drug-induced liver injury experience liver damage even at standard doses (Fontana et al., 2023). The current diagnostic approach for HILI remains a combination of medical history, laboratory parameters, detailed medication history, and/or the application of exclusionary diagnostic methods such as the Roussel Uclaf Causality Assessment Method (RUCAM)/the Revised Electronic Causality Assessment Method (RECAM), alongside liver biopsy (Hayashi et al., 2022; Zhao et al., 2024; Chalasani et al., 2021). Additionally, the prevalence of polypharmacy among most HILI patients in China, particularly the concurrent use of herbal/dietary fibre supplements alongside western medicines, complicates the diagnosis of HILI. This phenomenon is more common among the elderly population (Wang et al., 2018; Yu et al., 2024).

Although studies on drug-induced liver injury are frequently reported, to our knowledge, there remains a scarcity of systematic approaches to distinguish between patients with HILI, western medicine-induced liver injury (WILI), and non-drug-induced liver injury (NON-DILI) within the context of polypharmacy. Furthermore, analyses of potential risk factors within this patient cohort remain limited. Therefore, we have undertaken further exploration based on the identification of drug-induced liver injury. This study collected patients’ clinical characteristics, employed the RUCAM scale for causality assessment, and combined international authoritative databases (such as LiverTox Category A and B drugs) with drug package inserts to identify patients with WILI and HILI. Building upon this foundation, the study focuses on analysing risk factors among HILI patients, WILI patients, and NON-DILI patients. This aims to objectively explore the identification of risk factors in HILI patients and the implementation of risk prevention and control measures, thereby providing reference for clinical decision-making.

## 2 Patients and methods

### 2.1 Study population

This retrospective study analysed patients presenting with herb-induced liver injury at Nanjing Hospital of Chinese Medicine affiliated to Nanjing University of Chinese Medicine (NJHCM), between 01 January 2014 and 31 December 2023. The cohort was divided into a training set and a validation set to construct and validate a model identifying potential risk factors for herb-induced liver injury. Patients who developed liver injury following herbal medicine consumption at Jiangsu Province Hospital of Chinese Medicine (JSHCM) between 01 January 2024 and 01 March 2025 were incorporated into the test set to evaluate model performance.

Inclusion criteria: 1. Based on the clinical practice guidelines of the european association for the study of the liver (EASL) and the criteria proposed by the international consortium for critical care medicine for clinical chemistry (European Association for the Study of the Liver, 2019). 2. Compliant with the 2023 edition of the Guidelines for the Diagnosis and Treatment of Drug-Induced Liver Injury and the assessment criteria set forth in the Guidance for the Clinical Evaluation of Traditional Chinese Medicine-Induced Liver Injury issued by the China Food and Drug Administration. (1) Alanine aminotransferase (ALT) ≥5*the upper limit of normal (ULN); (2) Alkaline phosphatase (ALP) ≥2 times ULN; (3) ALT ≥3*ULN concurrently with total bilirubin ≥2*ULN (Mao et al., 2024; Xiao et al., 2019). 3. Patient records are complete and the RUCAM score can be calculated. 4. Patients with a RUCAM score of ≥6 were included in the study. (Note: The RUCAM scale categorises the causality between drugs and liver injury into five grades based on scoring results: Highly probable: >8 points; Probably: 6-8 points; Possibly: 3-5 points; Unlikely: 1-2 points; Can be excluded: ≤0 points.).

Exclusion criteria: 1. age <18; 2. Obstructive jaundice; 3. Sepsis; 4. Multiple organ failure; 5. The onset time was recent and the person died; 6. Liver damage caused by western medicine; 7. Acute viral hepatitis; 8. The information is incomplete.

The ethics committee of Nanjing Hospital of Chinese Medicine affiliated to Nanjing University of Chinese Medicine, approved this study and waived the requirement for informed consent. As this retrospective study was confined to researchers collecting existing data from cases for routine disease analysis. Data extracted from electronic medical records was coded to ensure confidentiality regters.

### 2.2 Research methods

First, retrieve data on patients with abnormal liver function where ALT, ALP, and total bilirubin exceed the upper limit of normal. Second, based on established inclusion and exclusion criteria, patients with elevated liver enzymes attributable to other clearly identified causes were excluded, thereby identifying those with unexplained liver enzyme elevation. Third, given the variation in latency periods for drug-induced liver injury across different medications and the absence of clear temporal boundaries, it is currently generally accepted that the latency period for specific drug-induced liver injury typically ranges between 5 and 90 days (Hoofnagle and Björnsson, 2019). Concurrently, the Guidelines for the Diagnosis and Management of Herb-Induced Liver Injury state that in patients presenting with elevated liver enzymes and suspected drug-induced liver injury, the median time to onset ranges from one to 3 months. Fourth, the RUCAM scale was employed to assess medication use in such patients during the preceding 3 months. Fifth, by referencing drugs classified as Category A (well-known cause) and Category B (highly likely) for hepatotoxicity in the LiverTox database, alongside western medicines documented in their package inserts as having confirmed hepatotoxicity, a list of western medicines associated with drug-induced liver injury has been compiled. Lastly, patients were grouped based on this drug list into suspected herb-induced liver injury and suspected WILI cohorts. Patients in the suspected HILI cohort with RUCAM scores ≥6 were classified as the positive group (HILI group); those in the suspected WILI cohort with RUCAM scores ≥6 constituted the western medicine control group (WILI group). Patients with a RUCAM score <3 (unlikely to be drug-induced) were assigned to the non-drug-induced liver injury control group (NON-DILI group). Our study did not include patients with unclear causality, whose scores fell within the 3-5 range. Additionally, the study compiled medication histories for all patients over the preceding 3 months, excluding preparations highly unlikely to cause liver injury (such as topical medications). All the above steps were independently verified by two researchers. In the event of disagreement, a third researcher was consulted to resolve the matter.

Retrieve medical records of patients with abnormal liver function, screen cases according to inclusion and exclusion criteria, and meticulously document demographic information, laboratory test results, medication details, and other relevant data. Information was collected via Microsoft Excel, with data reviewed and collated by two researchers. Variables with a missing percentage exceeding 25% were excluded. Variables with less than 25% missing values underwent multiple imputation using the “mice” package in R 4.5.1. To address multicollinearity, variance inflation factor (VIF) analysis was conducted using Python 3.11, with variables exhibiting VIF >5 being excluded.

### 2.3 Model development

**FIGURE 1:**
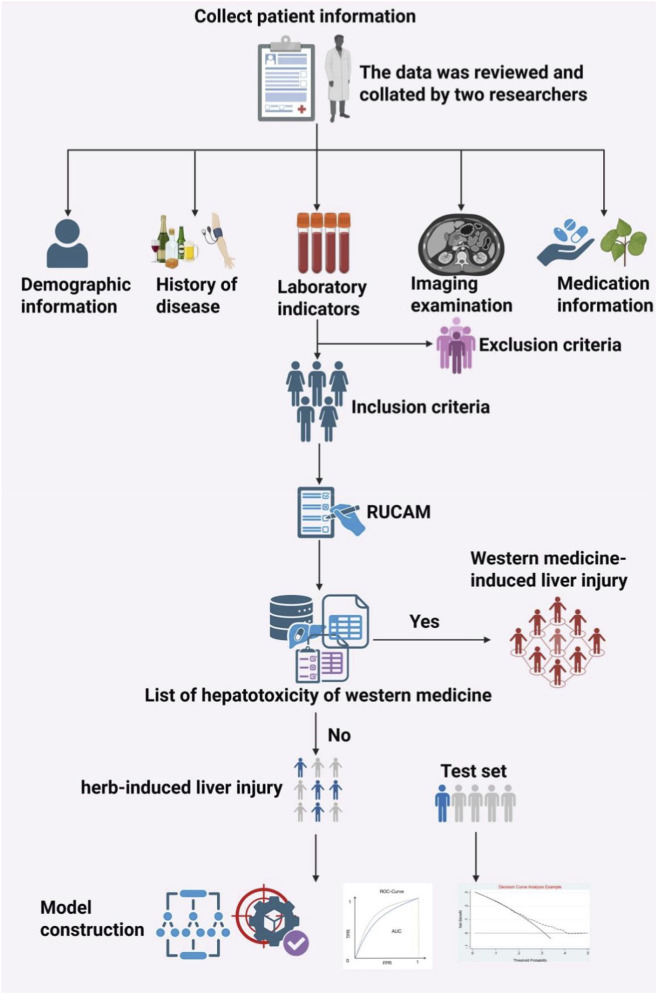
Research process for analysing risk factors for liver damage from herbal medicines.

First, adjust variables and their complexity through univariate analysis. Second, the binomial logistic regression analysis was employed to identify HILI predictors with *P* < 0.05. Third, using Python 3.11, patients from Nanjing Hospital of Chinese Medicine affiliated to Nanjing University of Chinese Medicine, were randomly divided into a training set and a validation set in a 7 : 3 ratio to mitigate potential overfitting issues. Furthermore, by incorporating patient data from other medical centres as a test set for validation. Evaluate the discriminatory capability of the binomial logistic regression analysis based on the area under the area under the receiver operating characteristic curve (AUC). The stability of the variable selection model was preliminarily assessed using the Hosmer-Lemeshow statistic (*P* > 0.05 indicates good agreement between predicted and actual values). Clinical net benefit was assessed using decision curve analysis (DCA), Figure 1.

### 2.4 Analysis of herbal medicine usage

To further clarify the association between herbal medicine types and liver injury categories in patients with herb-induced liver injury, this study describes and statistically analyses herbal medicines with RUCAM scores ≥6 among patients in the HILI group. Analyse the distribution of liver injury types (hepatocellular, cholestatic, mixed) induced by potentially hepatotoxic herbal medicines.

### 2.5 Statistical methods

Statistical analysis was conducted using IBM SPSS Statistics 27 and R 4.5.1. Quantitative data conforming to a normal distribution are expressed as mean ± standard deviation (Mean ± SD). Comparisons between groups were performed using Student's t-test (t-test). For non-normally distributed quantitative data, the median and interquartile range were employed for description. Intergroup comparisons were conducted using the Mann-Whitney U test (U test). Categorical data were described as percentages (%). Intergroup comparisons were analysed using the chi-square test or Fisher’s exact probability test to assess differences. *P* < 0.05 is considered statistically significant.

## 3 Results

### 3.1 Demographic characteristics and laboratory parameters

This study screened a total of 3,914 eligible patients from the NJHCM set and 3,334 eligible patients from the JSHCM set. Following the exclusion criteria, 1,694 and 2,225 cases were excluded respectively. Ultimately, 2,220 and 1,109 patients were included for RUCAM scoring respectively. Among the cases included in the NJHCM set, 230 cases had a RUCAM score <3 (NON-DILI group); 1,434 cases with 3 ≤ RUCAM ≤5; 556 cases with RUCAM ≥6; In the JSHCM set, 110 cases with RUCAM <3 were included (NON-DILI group); 666 cases with 3 ≤ RUCAM ≤5; 333 cases with RUCAM ≥6

**FIGURE 2:**
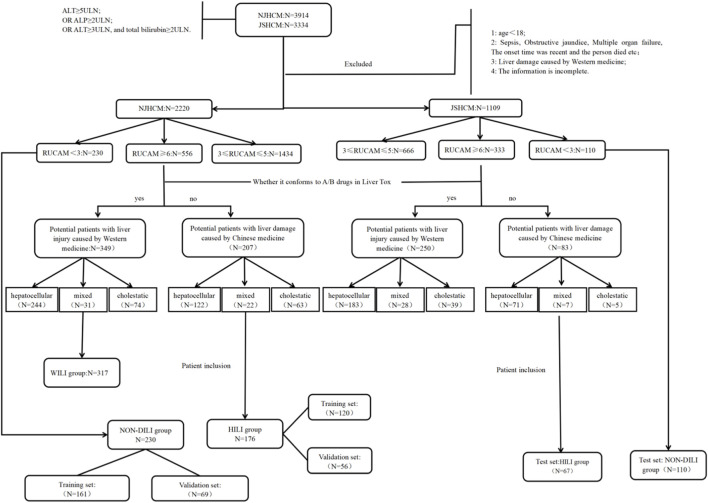
Hili patient inclusion and exclusion flowchart.

Using the western-induced liver injury drug list for grouping, the final NJHCM set included 176 patients with suspected herbal liver injury scoring ≥6 on the RUCAM scale (HILI group). This finding stems from an examination of the temporal relationships in medication use within this cohort, revealing that 31 patients had a history of taking potentially hepatotoxic western and herbal medicines. The RUCAM scores for these medications all fell within the grey zone of causality ambiguity, ranging from 3 to 5 points. Regarding the complex polypharmacy issues in such patients, there is currently no satisfactory method to distinguish whether liver injury stems from herbal medicines or hepatotoxic western pharmaceuticals. To minimise the impact of confounding factors on study outcomes, we excluded patients with particularly complex medication histories from enrolment. A total of 317 patients with RUCAM scores ≥6 were included in the suspected drug-induced liver injury group (WILI group). Thirty-two patients presented with complex polypharmacy issues that were challenging to assess. The JSHCM test set comprised 110 cases with RUCAM scores <3 (test set: NON-DILI group) and 67 cases with RUCAM scores ≥6 (test set: HILI group). Sixteen patients presented with complex polypharmacy issues that were difficult to determine conclusively Figure 2.

**FIGURE 3:**
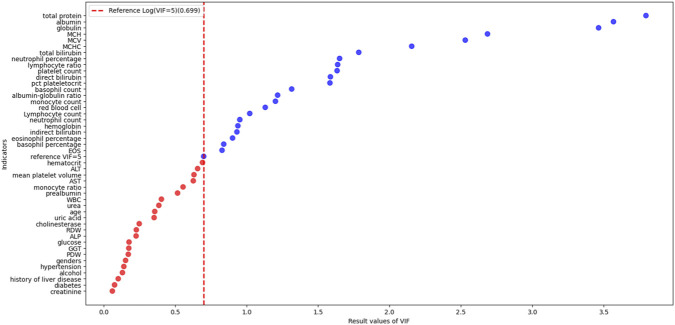
Scatterplot of VIF for preliminary screening indicators.

Following the collection of two sets of demographic data and laboratory test in-dicators, missing values were imputed using multiple imputation within the mice package in R 4.5.1. Multicollinearity was assessed via VIF testing, initially incorporating 45 indicators. Those without multicollinearity (VIF <5) were selected Figure 3. In patients with drug-induced liver injury, total bilirubin serves as a key indicator for assessing liver function and holds significant clinical importance (Xiao et al., 2019). Consequently, the study decided to incorporate it into subsequent analyses to explore its value as a risk factor. Final inclusion: hematocrit, ALT, mean platelet volume, aspartate aminotransferase (AST), monocyte ratio, prealbumin, total white blood cell count (WBC), urea, age, uric acid, cholinesterase, red blood cell distribution width (RDW), ALP, total bilirubin, platelet distribution width (PDW), gamma-glutamyl transferase (GGT), glucose, genders, hypertension, alcohol, diabetes, creatinine. Total of 22 indicators Table 1.

**TABLE 1:** VIF of candidate indicators.

Conditioning factor	VIF
Hematocrit	4.9034
ALT	4.5331
Mean platelet volume	4.2757
AST	4.2284
Monocyte ratio	3.5854
Prealbumin	3.2819
WBC	2.5303
Urea	2.4280
Age	2.2720
Uric acid	2.2493
Cholinesterase	1.7698
RDW	1.6895
ALP	1.6823
Glucose	1.5014
GGT	1.4959
PDW	1.4838
Genders	1.4202
Hypertension	1.3809
Alcohol	1.3505
History of liver disease	1.2617
Diabetes	1.1894
Creatinine	1.1483

### 3.2 Selection of predictive factors for HILI patients

Categorical variables were analysed using chi-square tests. Continuous variables were first tested for normality; depending on the results, either t-tests or Mann-Whitney U tests were selected. Only variables demonstrating statistically significant differences were ultimately included in the analysis Table 2.

**TABLE 2:** Demographic and baseline characteristics of patients in the HILI, WILI and NON-DILI groups.

Indicator	HILI group (n = 176)	WILI group (n = 317)	*P*	NON-DILI group (n = 230)	*P*
Age	64 (56,71)	71 (57,83)	<0.001	51 (33,65)	<0.001
Genders	​	​	0.206	​	0.560
Male	99 (56.3%)	185 (58.4%)	​	136 (59.1%)	​
Female	77 (43.7%)	132 (41.6%)	​	94 (40.9%)	​
Hypertension	63 (35.8%)	201 (63.4%)	<0.001	49 (21.3%)	0.001
Diabetes	21 (11.9%)	107 (33.8%)	<0.001	18 (7.8%)	0.164
History of liver disease	40 (23%)	69 (22%)	0.805	51 (22%)	0.895
Liver cyst	11 (28%)	16 (23%)	​	2 (4%)	​
Fatty liver	12 (30%)	29 (42%)	​	26 (51%)	​
Viral hepatitis	14 (35%)	24 (35%)	​	17 (33%)	​
Other liver diseases	5 (13%)	9 (13%)	​	8 (16%)	​
Alcohol	32 (18.2%)	48 (15.1%)	0.380	14 (6.1%)	<0.001
Laboratory indicators					
Prealbumin	175 (119,232)	171 (120,223)	0.434	188 (127,269)	0.080
AST	141 (69,242)	178 (99,383)	0.001	121 (68,205)	0.142
ALT	272 (93,411)	291 (221,454)	0.081	250 (75,358)	0.109
GGT	158 (76,371)	202 (91,343)	0.412	210 (81,374)	0.402
ALP	179 (110,315)	145 (89,284)	0.003	179 (93,318)	0.716
Total bilirubin	15.70 (10.02,29.10)	14.90 (9.40,30.05)	0.716	18.35 (11.90,36.75)	0.014
Cholinesterase	5077 (3100,7405)	344 (203,5709)	<0.001	5079 (340,8257)	0.409
Urea	5.44 (4.30,8.55)	6.64 (4.89,11.18)	<0.001	4.86 (4.08,6.08)	0.002
Creatinine	64 (51,83)	72 (56,101)	0.001	67 (59,79)	0.324
Uric acid	269 (192,360)	263 (186,382)	0.866	329 (260,392)	<0.001
Glucose	5.92 (5.24,7.40)	7.22 (5.62,9.64)	<0.001	5.81 (5.20,6.90)	0.485
Platelet distribution width	24.94 (16.10,52.35)	22.80 (15.75,53.85)	0.688	16.72 (16.10,26.28)	0.005
WBC	6.48 (4.70,8.90)	7.90 (6.00,11.10)	<0.001	6.40 (5.10,7.78)	0.329
Monocyte ratio	6.84 (5.32,8.83)	5.90 (4.60,7.55)	<0.001	6.23 (5.20,7.55)	0.009
Hematocrit	0.36 (0.32,0.40)	0.36 (0.31,0.40)	0.906	0.39 (0.35,0.43)	<0.001
RDW	13.7 (13.0,15.0)	13.7 (13.0,14.8)	0.781	13.6 (13.0,14.7)	0.222
Mean platelet volume	10.2 (9.3,11.3)	10.3 (9.4,11.2)	0.496	10.1 (9.4,10.9)	0.616

Data are expressed as median (interquartile range, IQR) or n (%).

To investigate potential risk factors among patients with herb-induced liver injury, western medicine-induced liver injury, and non-drug-induced liver injury, the HILI group was compared with the WILI group and NON-DILI group through risk factor analysis. Incorporate the statistically significant demographic characteristics and laboratory indicators identified in the univariate analysis into the binomial logistic regression analysis to screen for risk factors associated with the occurrence of HILI. Results indicate that ALP levels exhibited significant differences between the HILI group and WILI group (OR = 1.002, 95% CI: 1.001–1.004, *P* = 0.005), representing an independent risk factor distinguishing patients with herb-induced liver injury from those with western medicine-induced liver injury Table 3.

**TABLE 3:** Binary logistic regression analysis of univariate differences between HILI and WILI groups.

Indicator	B	Se	Wald	*P*	OR	95% confidence interval for OR	
						Lower limit	Upper limit
Age	0.002	0.008	0.083	0.773	1.002	0.987	1.018
Hypertension	−0.953	0.239	15.887	<0.001	0.386	0.241	0.616
Diabetes	−1.135	0.311	13.323	<0.001	0.321	0.175	0.591
AST	−0.001	0.000	5.788	0.016	0.999	0.998	1.000
ALP	0.002	0.001	7.877	0.005	1.002	1.001	1.004
Cholinesterase	0.000	0.000	47.597	<0.001	1.000	1.000	1.000
Urea	0.019	0.024	0.605	0.437	1.019	0.972	1.069
Creatinine	0.000	0.000	0.955	0.328	1.000	1.000	1.000
Glucose	−0.063	0.040	2.461	0.117	0.939	0.868	1.016
Total white blood cell count	−0.087	0.030	8.160	0.004	0.917	0.863	0.973
Monocyte ratio	0.053	0.037	2.083	0.149	1.055	0.981	1.134

Statistical analysis of the HILI group and NON-DILI group revealed six indicators associated with HILI occurrence: age, alcohol, urea, uric acid, platelet distribution width, and monocyte ratio. The Hosmer-Lemeshow test yielded a P value of 0.847 > 0.05, indicating satisfactory model fit. Based on the odds ratio (OR), the following five indicators suggest risk predictors for HILI patients: age (OR = 1.026, 95% CI: 1.010–1.042, *P* = 0.001), alcohol (OR = 4.645, 95% CI: 2.138–10.089, *P* < 0.001), urea (OR = 1.093, 95% CI: 1.026–1.164, *P* = 0.006), platelet distribution width (OR = 1.025, 95% CI: 1.012–1.039, *P* < 0.001) and monocyte ratio (OR = 1.254, 95% CI: 1.130–1.392, *P* < 0.001). Conversely, uric acid (OR = 0.996, 95% CI: 0.994–0.998, *P* < 0.001) was identified as a protective factor against HILI progression Table 4.

**TABLE 4:** Binary logistic regression analysis of univariate differences between HILI and NON-DILI groups.

Indicator	B	Se	Wald	*P*	OR	95% confidence interval for OR	
						Lower limit	Upper limit
Age	0.026	0.008	10.274	0.001	1.026	1.010	1.042
Hypertension	0.084	0.280	0.090	0.764	1.088	0.628	1.883
Alcohol	1.536	0.396	15.056	<0.001	4.645	2.138	10.089
Total bilirubin	−0.009	0.003	13.099	<0.001	0.991	0.986	0.996
Urea	0.089	0.032	7.596	0.006	1.093	1.026	1.164
Uric acid	−0.004	0.001	14.149	<0.001	0.996	0.994	0.998
Platelet distribution width	0.025	0.007	14.273	<0.001	1.025	1.012	1.039
Monocyte ratio	0.227	0.053	18.227	<0.001	1.254	1.130	1.392
Hematocrit	−2.014	2.058	2.247	0.134	0.046	0.001	2.581

### 3.3 Analysis of herbal medicine use in patients with herb-induced liver injury

A description of the herbal medicines consumed by 176 patients in the HILI group and the distribution of their liver injury types was undertaken. We analysed the ten most frequently used medicinal herbs among these patients Table 5. Pinellia ternata, Baical skullcap root, Chinese Thorowax root, Safflower, Indian buead, Alisma orientale, Scleromitrion diffusum (Willd.) R. J. Wang, Rhizoma Atractylodis, Rhubarb root and rhizome, Corydalis yanhusuo demonstrate distinctive advantages. The distribution of liver injury types induced by different herbal medicines predominantly exhibits hepatocellular liver injury.

**TABLE 5:** Top ten herbal medicines causing hepatic injury in the HILI group and distribution of injury types.

Herb	n (%)	Hepatocellular n (%)	Mixed n (%)	Cholestatic n (%)
Pinellia ternata	68	43 (63.24)	5 (7.35)	20 (29.41)
Baical skullcap root	53	38 (71.70)	3 (5.66)	12 (22.64)
Chinese thorowax root	21	15 (71.43)	0 (0.00)	6 (28.57)
Safflower	20	11 (55.00)	3 (15.00)	6 (30.00)
Indian buead	19	11 (57.89)	2 (10.53)	6 (31.58)
Alisma orientale	18	13 (72.22)	1 (5.56)	4 (22.22)
Scleromitrion diffusum (Willd) R J Wang	18	6 (33.33)	2 (11.11)	10 (55.56)
Rhizoma atractylodis	17	10 (58.82)	4 (23.53)	3 (17.65)
Rhubarb root and rhizome	16	8 (50.00)	1 (6.25)	7 (43.75)
Corydalis yanhusuo	16	11 (68.75)	1 (6.25)	4 (25.00)

### 3.4 Development of a risk model for HILI patients

**FIGURE 4:**
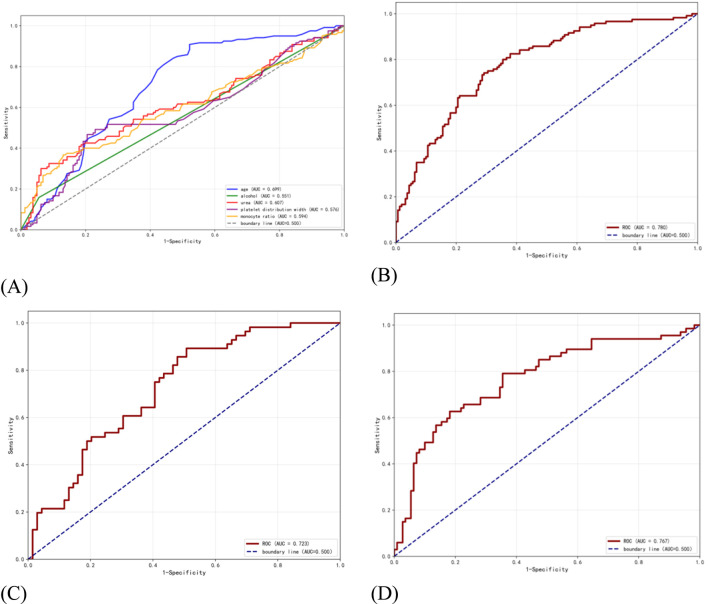
Auc performance evaluation of HILI risk factors. **(A)** Single risk factor indicator model **(B)** combined risk factor model of the training set **(C)** combined risk factor model of the validation set **(D)** ROC curve for the combined risk factor model of the test set.

**FIGURE 5:**
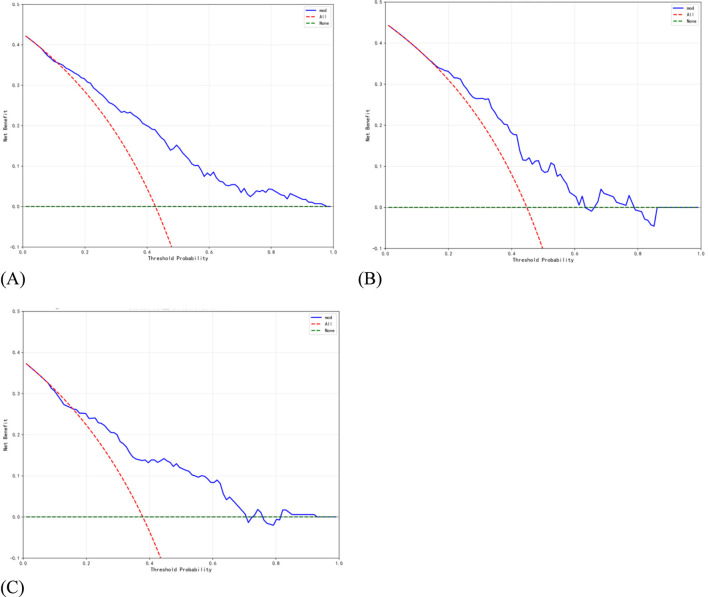
Dca performance evaluation of the combined risk factor model. **(A)** Combined risk factor model of the training set **(B)** combined risk factor model of the validation set **(C)** combined risk factor model of the test set.

To evaluate the applicability of independent risk factors for herb-induced liver injury and non-drug-induced liver injury. We analyse the performance of the individual risk factor variables age, alcohol, urea, platelet distribution width, and monocyte ratio within the training set. The results showed that the AUC values for age, alcohol, urea, platelet distribution width, and monocyte ratio were 0.699, 0.551, 0.607, 0.576, and 0.594 respectively. To compare the performance of individual risk factors with that of combined indicators, we constructed a combined indicator model based on the aforementioned five risk factors. The model achieved an AUC of 0.780, sensitivity of 0.741, specificity of 0.708, and threshold of 0.431 on the training set; Its performance demonstrated a marked improvement compared to the application of five individual risk factor variables. Additionally, the model achieved an AUC of 0.723 on the validation set, with a sensitivity of 0.892, specificity of 0.492, and threshold of 0.333; On the test set, it attained an AUC of 0.767, sensitivity of 0.626, specificity of 0.818, and threshold of 0.468 Figure 4. Given that the AUC metric of this joint indicator model did not exceed that of the training set in either the validation set or test set, or exceeded it by less than 10%, the model fitting may be considered successful (Muschelli, 2020). The DCA was employed to evaluate the clinical utility of the predictive model. The DCA curves for the training set, validation set, and test set all exceeded the two reference lines, indicating that the model possesses good clinical utility within the corresponding threshold range Figure 5.

## 4 Discussion

In this study, we examined patients presenting with liver injury following herbal medicine use and analysed their potential risk factors. By collecting basic information on this cohort and utilising a hepatotoxicity database, we aim to distinguish between HILI and WILI patients and identify potential risk factors among HILI patients. Analysis of the HILI and NON-DILI groups revealed a median age of 64 years for HILI patients and 51 years for NON-DILI patients. The odds ratio indicated that for each additional year of age, the risk of HILI occurrence increased by 2.6% (OR = 1.026, 95% CI: 1.010–1.042, *P* = 0.001). For middle-aged and elderly individuals, age constitutes a significant risk factor for inducing HILI, which may be closely associated with the diminished physiological regulatory capacity observed in this patient cohort and the complexity arising from multiple coexisting underlying conditions and polypharmacy (Yu et al., 2024; Lee et al., 2021). Research has found that alcohol increases the risk of liver damage, which may be related to either the direct toxicity of ethanol to the liver or the indirect toxicity of drug metabolites to the liver (Zhang et al., 2024). It is also possible that due to the effects of alcohol, the genetic diversity exhibited by patients, such as human leukocyte antigen (HLA), H-LA-C*01:02 and HLA-DRB1*09:01, is significantly associated with liver damage (Gopalakrishna et al., 2025). The close association between age and alcohol consumption factors and the occurrence of HILI has been confirmed by a substantial body of literature (Medina-Caliz et al., 2016; D Freire et al., 2023; Song et al., 2019; Jiang et al., 2021).

Previous studies have indicated that hypertension may serve as one of the potential risk factors for drug-induced liver injury (Park et al., 2021; Wang et al., 2025). This may be attributable to the fact that elderly patients with underlying conditions, particularly cardiovascular diseases, experience increased metabolic burden when using medications. Consequently, they are more susceptible to heightened hepatic stress and elevated risk of liver damage, a phenomenon that becomes particularly pronounced during prolonged or excessive administration of drugs metabolised by the liver (Yu et al., 2024; Baig et al., 2001). Whether such demographic characteristics may increase susceptibility to HILI requires further conclusive evidence to establish their association (Wang et al., 2018). In the univariate analysis of this study, hypertension exhibited a significantly higher prevalence in the HILI group (35.8% vs. 21.3%, *P* = 0.001). However, in the binomial logistic regression analysis adjusting for multiple confounding factors, hypertension was no longer an independent risk factor for HILI (OR = 1.088, 95% CI: 0.628–1.883, *P* = 0.764). To investigate whether this association was influenced by confounding factors such as age, we conducted further correlation analyses. The findings revealed that the disappearance of this association was primarily attributable to the confounding effect of age. Firstly, correlation analysis revealed a moderate linear association between age and hypertension prevalence (r = 0.413, *P* < 0.001). Secondly, age itself emerged as a significant independent predictor of HILI (OR = 1.026, 95% CI: 1.010–1.042, *P* = 0.001). This indicates that the association observed in univariate analysis is primarily attributable to the ageing of the HILI patient cohort, where elevated hypertension prevalence indirectly increases HILI risk. Consequently, the association between hypertension and HILI in this study more likely reflects age-related confounding effects rather than a direct causal relationship.

Declining organ function in patients increases the risk of hepatic injury. Lin HS (Lin et al., 2021) found that poor nutritional status in tuberculosis patients may be a significant factor contributing to hepatic injury in those undergoing anti-tuberculosis treatment. Our research further extends this understanding to patients with herb-induced liver injury. Research findings indicate that compared with the NON-DILI group, the HILI group exhibited lower baseline haematocrit levels at 0.36 versus 0.39 (*P* < 0.001). This may reflect impaired erythropoiesis in HILI patients, where deficiencies in haematopoietic precursors such as iron, vitamin B12, and folic acid lead to reduced hepatic synthesis of plasma proteins (Cheung et al., 2012). This state of nutritional deficiency directly impairs the liver’s metabolic and synthetic reserve functions. Furthermore, it may lead to anaemia and reduced systemic oxygen supply, placing hepatocytes in a state of hypoxia and ischaemia. This disrupts normal liver function and increases the risk of liver injury.

Results from the binomial logistic regression analysis comparing the HILI group with the NON-DILI group indicate that elevated baseline urea levels constitute a significant risk factor (OR = 1.093, 95% CI: 1.026–1.164, *P* = 0.006). This may suggest that malnutrition and hepatic cell dysfunction disrupt nitrogen metabolism and the urea cycle within the body, further impairing mitochondrial function and exacerbating hepatic inflammatory responses. Consequently, this leads to metabolic disorders in the liver and increases susceptibility to drug-induced liver injury (Gallego-Durán et al., 2022; Mihajlovic and Vinken, 2022). Furthermore, the analysis revealed a negative correlation between uric acid and HILI risk, suggesting it may exert a protective effect. This negative correlation may represent the body’s response during the initial stages of injury, where endogenous antioxidants such as uric acid are extensively depleted to counteract intense oxidative stress (El Ridi and Tallima, 2017). This combination pattern of elevated urea and reduced uric acid indicates that significant metabolic imbalance and heightened oxidative stress were already present within the body prior to the onset of HILI. However, total bilirubin, as an indicator for assessing hepatic dysfunction, did not emerge as a risk factor distinguishing patients with herb-induced liver injury from those with non-drug-induced liver injury (OR = 0.991, 95% CI: 0.986–0.996, *P* = < 0.001). Possible reasons include: 1. Clinical phenotype: Herb-induced liver injury predominantly manifests as hepatocellular liver injury (Xiao et al., 2019), primarily characterised by markedly elevated AST/ALT levels. However, other forms of liver injury, such as viral hepatitis, frequently involve elevated bilirubin due to cholestasis (Drexler et al., 2025; Kanda et al., 2025). Elevated bilirubin levels may be more readily apparent during the progression of other liver diseases. 2. Mechanism studies: The pathogenesis of most herb-induced liver injury involves metabolic activation products triggering mitochondrial toxicity, oxidative stress, or direct hepatocyte damage (Ma et al., 2023; Zhai et al., 2021). The association with obstructing bile ducts or disrupting bilirubin metabolic pathways is relatively weak.

This study analysed risk factors for liver injury caused by herbal and western medicines, identifying ALP as an independent risk factor for herb-induced liver injury (OR = 1.002, 95% CI: 1.001–1.004, *P* = 0.005). On the other hand, AST/ALT ratios, markers of hepatocyte injury, exhibited more pronounced elevations in the western medicine-induced liver injury group, Tables 2, 3, suggesting that western medicine-induced liver injury may more readily manifest as hepatocellular injury. Existing literature indicates that certain anti-tuberculosis and anti-tumour drugs may cause marked elevations in AST and ALT levels (Clinton et al., 2021), whilst statins such as atorvastatin are more likely to induce hepatocellular liver injury (Tillmann et al., 2019). Research into the phenotypic differences between herbal and western medicines in causing liver damage remains relatively limited at present. Whether western medicines are more likely than herbal medicines to induce a pattern of hepatocyte injury characterised by significant transaminase elevation requires further validation through subsequent studies.

In addition to metabolic indicators, liver damage may be reflected in blood cell parameters. This study demonstrates that patients in the HILI group exhibited significantly higher PDW compared to the control group (24.94 vs. 16.72), suggesting that elevated PDW may represent one of the risk factors for HILI. This phenomenon has been suggested by multiple clinical reports: Liu et al. indicated that thrombocytopenia is closely associated with the severity of drug-induced liver injury (Liu et al., 2022). Additional case reports indicate that patients experiencing thrombocytopenia following drug administration may develop severe cholestatic drug-induced liver injury (Yang K. et al., 2022). The reason may lie in the fact that the liver is the primary site for thrombopoietin synthesis. Liver injury can lead to decreased levels or activity of thrombopoietin, thereby causing alterations in platelet count and distribution width (Afdhal et al., 2008). Elevated PDW levels not only serve as a reference indicator for impaired hepatocyte function but may also contribute to microcirculatory disorders by affecting platelet function. This exacerbates hepatic ischaemia and hypoxia, thereby accelerating the progression of liver injury.

**FIGURE 6:**
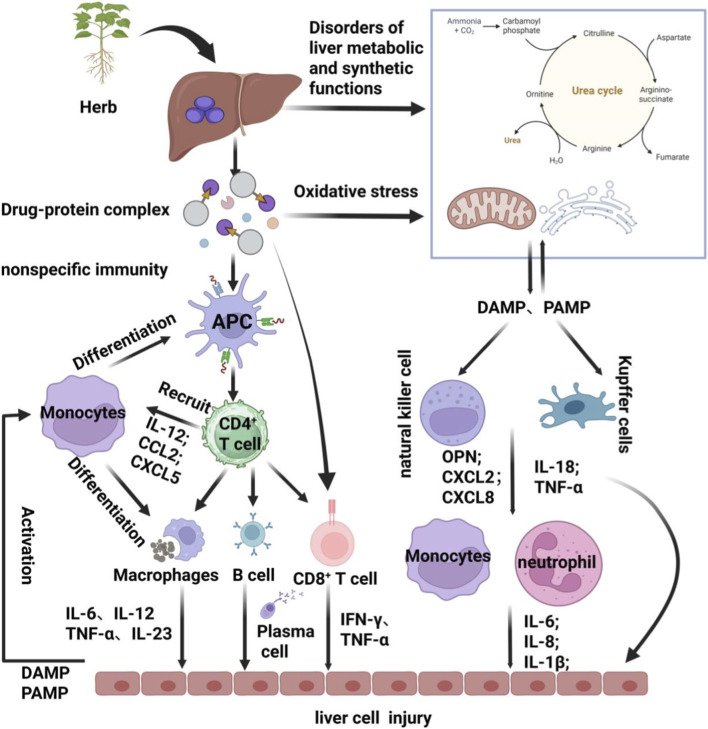
Schematic diagram of the HILI reaction mechanism.

The clinical manifestations and pathogenesis of HILI represent the combined outcome of multiple influencing factors, such as drugs, the host organism, and environmental conditions (Skat-Rørdam et al., 2025). The onset of HILI may originate from a pre-oxidative stress state induced by impaired liver function and drug metabolism, stemming from a weakened baseline liver condition due to systemic debilitation. This manifests as a precursor to hepatic dysfunction, signalled by imbalances in liver synthesis and metabolic functions. Ultimately, it is powerfully propelled by immune/inflammatory responses, primarily mediated by immune cells, completing the progression from susceptibility and initiation to full-blown onset Figure 6. This study found that patients in the HILI group exhibited a significantly higher baseline monocyte proportion compared to the NON-DILI group, suggesting that immune/inflammatory responses may play a pivotal role in the development and progression of HILI. The binomial logistic regression analysis indicated that the monocyte ratio was an independent risk factor for the HILI group (OR = 1.254, 95% CI: 1.130–1.392, *P* < 0.001). Moreover, the mechanisms by which various herbal medicines cause liver damage are closely associated with oxidative stress. Persistent oxidative stress in hepatocytes and stimulation by pro-inflammatory factors constitute one of the primary characteristics of drug-induced liver injury, during which hepatocyte damage and the expression of inflammatory markers significantly increase (Ma et al., 2023; Villanueva-Paz et al., 2021). Unlike other types of liver disease, the inflammatory infiltration in drug-induced liver injury is primarily mediated by T lymphocytes, particularly CD8^+^ T cells (Fontana et al., 2023). Plüß et al. (2022) and Hyun Yanget al (Yang et al., 2023) demonstrated the presence of activated CD8^+^ T cell infiltration in liver tissue from liver biopsy specimens of DILI patients, confirming that immune responses play a role in specific liver injury. However, an elevated monocyte count may serve as an indicator of systemic immune activation. These cells originate from haematopoietic stem cells in the bone marrow and rapidly migrate from the bloodstream to the liver upon stimulation by damage-associated molecular patterns (DAMPs) or pathogen-associated molecular patterns (PAMPs). Following infiltration, they may differentiate into macrophages. These cells act in concert with specifically activated T lymphocytes (such as CD4^+^, CD8^+^, etc.), releasing multiple injurious factors that ultimately result in hepatocyte damage (Lee et al., 2023).

This study, through an analysis of herbal medicines taken by HILI patients, found that the majority of these medicinal materials, when exerting their therapeutic effects in clinical applications, have been associated with risks of hepatotoxicity in the literature (Hsu et al., 2006; Li et al., 2021; Soldera, 2024). When assessing the risk of liver damage from herbal medicines, in addition to individual patient factors, the quality of the herbal materials may also be influenced by processing methods, production reserves and other factors, thereby increasing the risk of liver injury. Therefore, when patients take herbal medicines with potential high-risk hepatotoxicity, a comprehensive assessment system encompassing herbal medicine-organism-environment should be established. Strengthen quality control and post-administration liver function monitoring, and refine risk prevention measures including close observation, adjustment of treatment regimens, or even discontinuation of medication.

Our approach first identifies patients with WILI and HILI, then analyses risk factors among HILI patients relative to both the WILI group and the NON-DILI group. Construct risk factor models for patients with HILI and non-drug-induced liver injury, evaluate the clinical applicability of these models using AUC and DCA, and ultimately enable focused monitoring of high-risk groups. HILI risk factor analysis provides crucial real-world evidence for the early identification of this patient cohort, recommending regular follow-up for high-risk individuals. This approach transforms patients with herb-induced liver injury from a state of uncertainty to one where risk is manageable.

Although our research has identified potential risk factors for HILI, our study has several limitations when focusing on risk factor screening. (1) The patients in this study were sourced from dual-centre clinical data, with limitations in target patient enrolment. To further optimise the model’s performance, its robustness requires validation through the inclusion of data from additional medical centres. (2) Patients undergoing treatment typically receive both traditional chinese medicine and western pharmaceuticals concurrently. Although our methodology has preliminarily distinguished patients with western drug-induced liver injury from those with herb-induced liver injury, the multi-component nature of chinese herbal formulations renders the analysis complex and challenging. (3) Laboratory test indicators were constrained by the retrospective nature of the study, with significant gaps in data regarding other parameters assessing hepatic metabolic dysfunction and immune dysfunction in the subjects’ laboratory tests. Consequently, we were unable to incorporate potential risk factors for HILI patients into our analysis.

## 5 Conclusion

This study identified patients with HILI and analysed them alongside the WILI group and the NON-DILI group. It was found that ALP may be an independent risk factor distinguishing patients with herb-induced liver injury from those with western-medication-induced liver injury. Potential risk factors for herb-induced liver injury and non-drug-induced liver injury patients: age, alcohol, urea, platelet distribution width and monocyte ratio were incorporated to construct a risk factor model. The model’s discriminatory capability and clinical efficacy were validated through AUC analysis and decision curve analysis. In clinical practice, particular attention should be paid to monitoring liver function in elderly patients with risk factors, assisting clinicians in medication decisions to minimise the risk of herb-induced liver injury. Future research into the underlying mechanisms should place greater emphasis on exploring the deeper interactions between hepatic metabolic dysfunction and concomitant immune dysregulation, thereby enabling the development of targeted prevention and treatment strategies.

## Data Availability

The raw data supporting the conclusions of this article will be made available by the authors, without undue reservation.
